# Polysomnographic Profile and Clinical Phenotypes of Osa in Association with Residual Sleep Apnea in Children

**DOI:** 10.3390/life15081282

**Published:** 2025-08-13

**Authors:** Alžbeta Soršáková, Anna Durdikova, Peter Durdik, Jakub Sulov, Dominika Dvorska, Peter Banovcin, Milos Jesenak

**Affiliations:** Department of Pediatrics and Adolescent Medicine, Jessenius Faculty of Medicine in Martin, Comenius University in Bratislava, University Hospital Martin, 036 59 Martin, Slovakia; betmajtanova@gmail.com (A.S.);

**Keywords:** residual obstructive sleep apnea in children, phenotypes of OSA in children, standard overnight polysomnography

## Abstract

**Introduction**: Residual obstructive sleep apnea (OSA) is defined as persistence of the AHI at ≥1 respiratory event per hour of sleep after otorhinolaryngology intervention in pediatric population. In terms of OSA phenotypes in children, we recognize the common, adult, and congenital phenotypes. **Material and Methods:** We studied 34 pediatric patients with OSA diagnosed by standard overnight polysomnography (PSG). Based on the results, all 34 patients were indicated for adenotonsillectomy (ATE). After 3 months, patients were invited to the sleep laboratory for second control PSG. These OSA patients were divided according to clinical phenotype into two groups: common phenotype and adult phenotype. **Results:** Residual OSA was diagnosed in 24 patients with a mean AHI 6.67 ± 9.17 after ATE procedure. Incidence of severe residual OSA was 17.6%; moderate residual OSA was 20.6%; mild residual OSA was 32.4%. Residual OSA in classic phenotype was present in eight patients (57.1%). In total, 16 patients with the adult phenotype of OSA (80.0%) were diagnosed with residual OSA. Prevalence was significantly higher in children with the adult phenotype (*p* < 0.001). **Conclusions:** We concluded that there is a higher risk of residual OSA in children with the adult phenotype. Phenotyping of children with OSA appears to be an important tool for further management and also in predicting the severity of residual OSA.

## 1. Introduction

Pediatric obstructive sleep apnea (OSA) is a childhood sleep related breathing disorder. It is defined by an upper airway dysfunction, which causes complete or partial airway obstruction during sleep, leading to decreased oxygen saturation or arousals from sleep. It can have an influence on childhood behavior, neurodevelopment, metabolism, and overall health. Early recognition and evaluation lead to proper treatment and prevention of long-term consequences [1].

Due to both intrinsic and extrinsic factors there is an increased risk of an abnormal collapse of the upper airway. Critical pressure in the airway is needed to maintain patency, and the intrinsic factors are based on it. To the extrinsic factors belong fat deposits, hypertrophy of tissues, and craniofacial anomalies that stray from normal anatomy, which contribute to an increased incidence of airway collapse [2].

The prevalence of OSA in preschool children has increased over the past decade. Studies published before 2014 reported prevalence rates ranging from 3% to 9.4%. However, more recent research from 2016 to 2023 indicates higher prevalence rates, between 12.8% and 20.4% [3].

According to the American Academy of Sleep Medicine (AASM), the clinical diagnostic criteria for pediatric OSA include the presence of one or more of the following symptoms: snoring; labored, paradoxical, or obstructed breathing during sleep or drowsiness; hyperactivity; behavioral problems; and learning disabilities or other cognitive problems. Other possible clinical findings of pediatric OSA include night sweats, nocturnal enuresis (especially secondary), headaches on awakening, mouth breathing (during sleep or while awake), tonsillar hypertrophy, adenoidal facies, micrognathia/retrognathia, and a high-arched palate.

A polysomnography (PSG) profile for pediatric obstructive sleep apnea includes an obstructive apnea-hypopnea index (AHI) of ≥1 event per hour of sleep, with or without the pattern of obstructive hypoventilation-defined hypercapnia during ≥25% of the total sleep time, together with snoring, flattening of the inspiratory nasal pressure waveform, or paradoxical thoracoabdominal movement.

Management approach in pediatric OSA is defined with help from the evaluation of respiratory events according to the polysomnography findings. The AHI is known and used to assess the severity of obstructive sleep apnea. When the AHI is less than five events per hour, OSA is classified as mild. An AHI between 5.0 and 9.9 indicates moderate OSA, while an AHI of 10 or higher suggests severe OSA [4].

In OSA, phenotyping involves categorizing patients into smaller, more uniform groups (phenotypes) using clinical, pathophysiological, cellular, or molecular features. Recent studies suggest that this strategy aids in comprehending the diversity of OSA, which presents with varying symptoms, root causes, associated conditions, and therapeutic results.

In terms of OSA phenotypes in children, we identify common, adult, and congenital phenotypes [5]. They may overlap with each other [6].

In children with OSA, obesity is a prominent feature of the adult phenotype, often accompanied by a short neck, fat deposits around the neck, and midfacial hypoplasia [7]. While many children with OSA generally present with enlarged tonsils, adenoids, or obesity, certain subgroups with existing conditions also face an increased risk [8]. Pediatric OSA exhibiting adult characteristics is closely linked with obesity and positional sleep apnea, impacting up to 60% of obese children, especially older ones with a higher BMI.

This condition leads to frequent sleep disturbances and severe nighttime hypoxemia, potentially resulting in neurocognitive and cardiovascular complications if not promptly treated. Children with positional OSA experience higher AHI scores when lying supine and fewer respiratory incidents in other positions [9,10].

Enlarged tonsils and/or adenoids, a long face, a narrow palate, or mild malocclusion are characteristic features for the common phenotype. The incidence of pediatric OSA is the highest between 3 and 6 years of age. There is a coincidence with the typical period of tonsil and adenoid enlargement [11]. The partial or complete occlusion of the nasal and oropharyngeal airways during sleep is caused by tonsil and/or adenoid hypertrophy leading to airway compromise and respiratory distress in children [12]. There is a presence of alterations in sleep architecture parameters in the common form of pediatric OSA.

Sleep cyclization and stage distribution remained stable, with no notable differences observed in SPT, TST, WASO, stage R, stage N2 sleep, and sleep efficiency between those with and without OSA. Children with OSA had reduced stage N3 sleep and increased stage N1 sleep compared with controls. OSA frequently manifests as a stage R sleep phenomenon. Although the duration of stage R did not experience a significant change, there were more respiratory arousals [13].

The congenital phenotype of pediatric sleep apnea manifests with OSA symptoms from infancy, and patients may have a history of prematurity. Patients may also be associated with conditions such as Down syndrome, neuromuscular disorders, and craniofacial anomalies commonly associated with the Pierre Robin sequence. These conditions include micrognathia or retrognathia, which increases the risk of upper airway obstruction during sleep [14]. Children with congenital syndromes, craniofacial abnormalities, and/or neuromuscular disorders show a notably higher risk of OSA than those without such conditions. Prevalence rates are reported to range from 45% to 76% in Down syndrome, reach up to 48% in Turner syndrome, account for 15% in cerebral palsy, escalate to 85% in the Pierre Robin sequence, and surpass 50% in cases of achondroplasia [15,16,17,18].

Numerous genetic and congenital syndromes have been linked to the congenital phenotype of pediatric OSA, with the clinical presentation in these patients frequently exhibiting high variability. In these instances, the therapeutic impact of adenotonsillectomy can be complicated by existing comorbidities or intricate anatomical and functional irregularities, making it difficult to accurately evaluate the results of the surgery. Consequently, patients with syndromic OSA were not included in this study.

The first line therapy and most effective treatment method so far in uncomplicated pediatric patients with adenotonsillar hypertrophy and OSA of moderate and severe degree is ATE. ATE can also serve as an initial treatment for children with multifactorial OSA, involving factors like obesity, when there is significant adenotonsillar tissue. While ATE generally leads to improvement in OSA for obese children, the results might be less favorable compared with those observed in children of normal weight [19].

According to the latest recommendations, the surgery itself should be preceded by diagnostic PSG [20,21]. The effect of the operation in the form of improvement of clinical symptoms should be visible 3 to 6 months after the operation, which is considered the right time to perform a follow-up PSG [6,22].

Residual OSA is considered to be the persistence of 1 or more respiratory events in the form of obstructive apnea or hypopnea per hour of sleep, on a control PSG examination of a pediatric patient, after ATE has been performed. Recent studies reveal that 13–29% of non-obese children with uncomplicated OSA and up to 73% of obese children continue to exhibit residual OSA after ATE. Risk factors for residual OSA include obesity, severe preoperative OSA (AHI ≥ 10/h), genetic syndromes (such as Down syndrome), craniofacial abnormalities, neuromuscular disorders, recurrent upper respiratory infections, and asthma [23,24,25,26].

In our work, we addressed the issue of residual OSA in children, which is still an open, topical, and debated topic in the field of modern pediatric sleep medicine.

## 2. Materials and Methods

This study is a retrospective analysis involving a follow-up of 154 patients who were assessed in the pediatric sleep laboratory at the Jessenius faculty of Medicine, University hospital Martin, for suspected breathing disorders during the period from September 2020 through to September 2022. In this cohort, 34 patients were diagnosed with OSA, underwent standard overnight PSG examination prior to the procedures, and had surgical intervention as well as a subsequent follow-up PSG examination with an interval of 3–6 months post-procedure. All patients underwent comprehensive evaluation, including detailed clinical and sleep history, physical examination, and confirmation of OSA by overnight polysomnography demonstrating obstructive events.

Patients were categorized into two phenotypic cohorts predicated on clinical manifestations and anthropometric parameters.

The common phenotype predominantly manifests in otherwise healthy children aged between 2 and 8 years, maintaining a normal body mass index (BMI) in relation to age and sex. Qualified participants demonstrated a consistent clinical profile characterized by habitual loud snoring accompanied by breathing pauses or episodes of gasping during sleep and restless sleep with frequent positional changes, occasionally adopting unusual sleep postures such as sleeping with the head tilted backward or in a semi-upright position. Physical examination typically revealed adenoidal facies, marked by a long face, open-mouth posture, and a high-arched palate, in conjunction with hypertrophy of the adenoids and/or palatine tonsils.

The adult phenotype of pediatric OSA is more frequently identified in adolescents and older children. This phenotype differs from the common presentation of pediatric OSA by its association with obesity, metabolic risk factors, and less pronounced adenotonsillar hypertrophy. Eligible individuals were typically over the age of 8 years and presented with a body mass index (BMI) above the 95th percentile for age and sex, consistent with pediatric obesity. Physical examination findings include central obesity, a large neck circumference, and signs of upper airway narrowing.

Clinically, these patients frequently reported habitual snoring, witnessed apneic episodes, gasping or choking during sleep, and non-restorative or fragmented sleep. Daytime symptoms included excessive sleepiness, poor academic performance, behavioral or mood disturbances, and decreased attention span. Many exhibited features suggestive of underlying metabolic dysregulation, such as insulin resistance or elevated blood pressure.

Patients with genetic syndromes, craniofacial abnormalities, neuromuscular diseases, and allergies were excluded from the study to avoid potential bias. The study focused on comparing children with the common and adult phenotypes of obstructive sleep apnea (OSA). Children presenting with the congenital OSA phenotype were not included in the analysis, as they typically suffered from multiple comorbidities or varying degrees of respiratory insufficiency. These conditions often affected both the feasibility and course of surgical treatment and would have introduced significant confounding factors into the analysis. Additionally, children with structural or functional abnormalities in the otorhinolaryngological region such as nasal or oropharyngeal deformities were not included. Patients diagnosed with vasomotor rhinitis, allergic rhinitis, and asthma or those with a current or recent respiratory tract infection were also excluded. These exclusion criteria were strictly applied to preserve the purity of the sample and ensure the reliability of the study findings. None of the patients were undergoing pharmacological treatment for sleep apnea or any other respiratory conditions. Individuals with a history of previous otorhinolaryngological interventions were excluded from the study.

All children were evaluated in our pediatric sleep center by means of a full-night video PSG measurement following an adaptation night in the hospital. Standard overnight PSG recordings were obtained by means of ALICE 6 LDx (Phillips Respironics, Murrysville, PA, USA). The variables recorded included an electroencephalogram with at least eight channels, an electro-oculogram, a submental electromyogram, and an electrocardiogram. Sleep stages were scored according to the standard criteria of the American Academy of Sleep Medicine (AASM). Chest and abdominal movements were measured by strain gauges. Oronasal airflow was recorded with a thermocouple and, where the child tolerated a nasal cannula, a nasal pressure monitor. Peripheral oxygen saturation was monitored with a pulse oximeter (Masimo Pulse Oximeter Finger Sensor, Masimo, CA, USA). Central, obstructive, and mixed apnea events, respiratory-related arousals (RERA), and hypoventilation during sleep were counted according to the criteria established by the AASM.

In our cohort OSA was defined by an obstructive apnea/hypopnea index (OAHI) greater than or equal to 1 respiratory event per hour. Severity of OSA was classified based on OAHI values as follows:

Mild OSA: OAHI ≥ 1 and <5 events/h
Moderate OSA: OAHI ≥ 5 and <10 events/h
Severe OSA: OAHI ≥ 10 events/h

Central sleep apnea (CSA) was defined by a central apnea index (CAI) greater than or equal to 5 events per hour. These thresholds align with established pediatric sleep medicine guidelines and reflect clinically meaningful distinctions in disease severity [4]. Residual OSA was defined by an obstructive apnea/hypopnea index (OAHI) greater than or equal to 1 event per hour [26].

All polysomnographic recordings were independently reviewed, evaluated, and manually scored by a qualified investigator (A.D.), applying visual pattern recognition techniques in accordance with the most recent scoring criteria established by the American Academy of Sleep Medicine (AASM). The scoring process adhered strictly to standard epoch-based guidelines and included classification of sleep stages, respiratory events, arousals, and limb movements based on established visual features.

Various parameters were studied, including Sleep Period Time (SPT), Time in Bed (TIB), Total Sleep Time (TST), Wake After Sleep Onset (WASO), sleep latency, Rapid Eye Movement (REM) latency, quantity of REM, sleep efficiency, effectiveness of deep sleep, percentage of specific sleep stages, duration within each sleep stage, arousal index due to respiratory reactions, limb movements, spontaneous awakening reactions, and respiratory events such as central apnea, obstructive apnea, hypopnea, Apnea-Hypopnea Index (AHI), saturation levels (average, maximal percentage of desaturation, and saturation for each sleep phase), snoring, stage of obstructive sleep apnea, as well as any additional treatment.

In total, 68 polysomnographic records were analyzed. Patients were divided into phenotypic groups based on clinical features such as age, weight, height, Body Mass Index (BMI), percentiles, and adenotonsillar hypertrophy. Furthermore, we evaluated basic anthropometric parameters, selected polysomnographic parameters, and assessed treatments in these patients.

The study sample consisted of 29 male participants and 5 female participants, with a mean age of 9.2 ± 4.4 years, an average Body Mass Index z-score of 1.42 ± 1.62, and an average AHI of 32.2 ± 31.9 respiratory events per hour of sleep before surgery. After surgery, the BMI z-score was 1.67 ± 1.41.

The common phenotype, characterized by adenotonsillar hypertrophy and typical adenoid facies, includes a non-sleeping elongated face, mouth breathing, and rhinolalia. This common phenotype was identified in 41.2% of the patients in our cohort.

Average age in the common phenotype was 6.1 ± 3.1 years. The AHI before surgery was 40.2 ± 34.9 respiratory events per hour of sleep.

Representation in terms of sex in the common phenotype was present in 14.3% of females and 85.7% of males. The preoperative BMI z-score was −0.29 ± 0.83. The BMI z-score after surgery was 0.20 ± 0.74.

The adult phenotype, characterized by obesity and fat accumulation in the neck region, was observed in approximately 58.82% of the participants. Representation in terms of sex in the adult phenotype was present in 15% of females and 85% of males. The gender distribution for each phenotype is shown in Table 1. Patients exhibiting the adult phenotype were significantly older (*p* < 0.001) compared with those with the common phenotype, with a mean age of 11.41 ± 3.8 years. Additionally, the BMI z-score was markedly higher in the adult phenotype group (*p* < 0.001) with an average BMI z-score of 2.61 ± 0.67. After surgery, the BMI z-score was 2.70 ± 0.62. The AHI before surgery was 27.1 ± 20.5 respiratory events per hour of sleep in adult phenotype. The association between age, z-score, AHI, and phenotypes is demonstrated in Table 2.

Data from the observational study were statistically analyzed using Microsoft Excel 365. The variables were normally distributed tested by the Shapiro–Wilk test, so they were presented as mean and standard deviation. Descriptive statistics were applied to all variables to describe demographic and anthropometric parameters. To test the significance of hypotheses, the chi square test and Student’s *t*-test were applied. The Pearson correlation coefficient was used to correlate the association of the BMI z-score and the severity of residual OSA. In all analyses, a *p*-value of <0.05 was interpreted as the level of statistical significance. Correlation coefficients r = 0–0.19 were considered as weak, 0.20–0.39 as mild, 0.40–0.59 as moderate, 0.60–0.79 as moderately strong, and 0.80–1.0 as a strong correlation.

## 3. Results

### 3.1. Residual OSA

Residual OSA was present in 70.6% of patients with a mean AHI value of 6.67 ± 9.17 respiratory events per hour of sleep in patients after surgery. The average age was 9.2 ± 4.4 and average AHI was 32.2 ± 31.9. Mild residual OSA was present in 32.14% of patients. In the case of 20.6% of patients, moderate OSA was present. Severe residual OSA persisted in 17.6% of patients. In the group of children with the common phenotype, where obesity was not present, the incidence of residual OSA was 57.1% with a mean AHI of 6.96 ± 7.94 respiratory events per hour of sleep. In the group of children with the adult phenotype and obesity predominating as a phenotypic feature, up to 80% of patients with residual OSA were present with a mean AHI of 10.39 ± 10.72 respiratory events per hour of sleep. Table 3 summarizes the incidence of residual OSA across different phenotypes based on our own analysis.

Prevalence was significantly higher in children with the adult phenotype (*p* < 0.001). The apnea/hypopnea index value before ATE had no significant effect on the incidence of residual OSA after surgery (r = −0.049).

It was found that the common phenotype group experienced a more significant increase in their z-score (by +0.49) after ATE in comparison with the adult phenotype group who changed their weight only slightly. The BMI z-score showed no association with the risk of OSA severity before ATE across the entire cohort, the common phenotype (r = −0.150) (*p* > 0.05) 95% CI (–0.630 to 0.413) and the adult phenotype (r = −0.033) 95% CI (−0.469 to 0.416) (*p* > 0.05) After ATE, there was no association with the risk of OSA severity as well, in the common phenotype (r = −0.120) 95% CI (−0.612 to 0.439) (*p* > 0.05) and adult phenotype (r = −0.14) 95% CI (−0.549 to 0.323) (*p* > 0.05).

There was no significant difference in the sex distributions between the common and adult phenotypes (*p* = 0.82).

### 3.2. Further Management

In our patient cohort, we can see that only 29.4% of patients had an effective ATE, so much so that there was not even one respiratory event in the form of obstructive apnea or hypopnea per hour of sleep. On the contrary, 17.6% of patients were still in the severe residual OSA group after ATE; in these patients, ventilatory support was required in further treatment and in the form of CPAP and BiPaP. Table 4 presents a comprehensive comparison of OSA severity prior to and following surgical intervention, demonstrating alterations in the distribution of severity categories within the cohort.

## 4. Discussion

Unresolved OSA in children following ATE removal, referred to as residual, is becoming an increasing concern in the pediatric population. This condition is dependent on various factors, which often overlap. Risk factors for persistence include obesity, severe OSA prior to surgery, specific age groups, as well as certain complex diseases such as genetic disorders [27].

ATE remains one of the most common surgical interventions performed in the pediatric population. The efficacy of ATE in the treatment of OSA can be assessed using objective data derived from PSG both before and after the operation. According to our study, in 29.4% of patients, ATE was so effective that at the second follow-up PSG there was a correction of the apnea hypopnea index to values under 1.

Previous partial otorhinolaryngological surgeries performed before complete adenotonsillectomy could have influenced the outcomes and complicated the evaluation of the independent impact of adenotonsillectomy. Such prior interventions might have altered the anatomical baseline and physiological response, thereby affecting the postoperative assessment and confounding the interpretation of our results. Therefore, patients with a history of prior otorhinolaryngology intervention were excluded from the study.

Anomalous PSG findings indicative of persistent OSA have been documented in approximately 20–40% of patients, with a higher prevalence observed in those with severe manifestations [28].

Our study revealed that 70.6% of the patients had residual OSA, and it did not include those with risk factors such as craniofacial anomalies, neuromuscular diseases, asthma, allergies, and genetic syndromes. The prevalence of residual OSA was higher in older children, particularly in the adult phenotype group, where obesity was more common. This finding aligns with previous studies suggesting that obesity is a key risk factor for persistent OSA, even after surgical treatment.

Persistence of OSA after ATE is notably higher in obese children. Several studies have demonstrated that obesity is a major risk factor for the recurrence of OSA following surgery [27,29,30,31].

All children with OSA who have undergone otorhinolaryngology intervention surgery should be monitored for residual symptoms. PSG should be performed to identify persistent disease [23]. This highlights the importance of a comprehensive postoperative assessment, which should include follow-up polysomnography to identify individuals who may remain at risk for ongoing OSA. The important limitation is related to follow-up compliance. In our study, parental non-compliance also played its role, with patients often arriving for follow-up late or not at all, after the wrong procedure had been performed, or when symptoms reappeared. In clinical practice, we observed that parents often returned with their children for evaluation only when symptoms reappeared or worsened, rather than at scheduled follow-up visits. This pattern may have introduced bias into our assessment of treatment outcomes, as asymptomatic or improved cases were less likely to be captured, potentially skewing the observed incidence of residual or recurrent OSA.

The education of parents and patients is identified as an important key, and it should be used with clinical and decision support [32].

Children with higher BMI z-scores often present with additional upper airway obstructions, including fatty tissue deposition around the neck and pharyngeal muscles, which exacerbate the risk of airway collapse during sleep [13]. Our study aligns with these findings, where the adult phenotype (characterized by obesity) showed a significantly higher prevalence of residual OSA (80%) compared with the common phenotype (57.1%). Obesity-related factors may impede the effectiveness of adenotonsillectomy. Although no association was found between the preoperative BMI z-score and the severity of residual OSA postoperatively, future research should explore the role of changes in anthropometric parameters in the development of residual OSA.

Kalra et al. (2005) and Verhulst et al. (2009) confirm that there is a significant improvement of AHI after weight adjustment [32,33]. According to Ehsan et al. (2024) [34], there is a strong association between obesity and OSA in children. Children with obesity and residual OSA after ATE should be encouraged to lose weight and offered support to assist these efforts [35].In such cases, additional management strategies, including CPAP (continuous positive airway pressure) or BiPAP (bilevel positive airway pressure), may be required to mitigate residual OSA symptoms. Positive airway pressure therapy involves the utilization of a device that generates positive airway pressure at variable levels, quantified in centimeters of water pressure. It is imperative for the PAP machine to deliver pressures that exceed the critical closing pressure of the upper airway to maintain an open airway throughout the breathing cycle [22,36].

CPAP therapy is often effective for managing mild to moderate OSA; it remains an essential tool in children with persistent or severe OSA, especially in those with obesity [35,37]. In our cohort, 8.82% of patients with residual OSA required CPAP. For instance, where residual OSA remains post-surgery or in cases where surgical intervention is not indicated. PAP therapy proves to be effective and safe. CPAP is linked to important clinical benefits, including decreased insulin resistance, diminished cardiovascular disease risk, and an improvement in behavioral concerns in children [34]. In our cohort, in 17.6% of patients, an apnea/hypopnea index of 10 or more persists. Therefore, postoperative monitoring and individualized care plans are essential for optimal management.

Numerous studies have confirmed that sleep architecture in pediatric OSA is significantly disrupted. According to Durdik et al., sleep cyclization and the distribution of sleep stages were retained in the common phenotype among children with OSA. They observed notably shorter durations of stage N3 sleep in children with the common phenotype of OSA compared with the control group, while stage N1 sleep was considerably prolonged [13], as per Fosland there is a reduction in deep sleep (N3) and rapid eye movement (REM) sleep [38]. These disruptions in sleep quality highlight the importance of addressing OSA to prevent long-term cognitive and behavioral issues.

The significance of ongoing follow-up in children who have had an ATE for OSA is vital. Although the postoperative resolution of symptoms such as snoring and mouth breathing can occur, residual OSA might still exist, notably in children with obesity or other risk factors. Follow-up PSG is essential for identifying any persistent OSA and determining if further intervention is necessary [21,28]. While PSG results are key to identifying the existence and intensity of OSA, they do not show the exact anatomical cause or location of the obstruction. Awake flexible endoscopy can help to evaluate specific anatomic obstructions like lingual tonsil enlargement or regrowth of adenoids; nevertheless, awake examinations do not accurately reflect the patient’s airway conditions during sleep [39].

The DISE (drug induced sleep endoscopy) technique entails a comprehensive evaluation of the upper airway using a flexible endoscope while patients are in a pharmacologically induced sleep-like state. The endoscope is inserted through the nostrils to inspect the nasopharynx, oropharynx, larynx, and in certain instances the trachea [40].

As advancements in pediatric sleep medicine progress, innovations such as DISE are anticipated to facilitate more individualized treatment strategies, thereby improving outcomes for children with both obstructive and residual sleep apnea. The significance of DISE in pinpointing precise sites of airway obstruction is vital for targeted interventions for children with residual OSA. This technique is particularly advantageous in instances where the etiology of residual OSA is ambiguous, assisting clinicians in planning for potential subsequent surgical procedures or alternative therapeutic interventions [41].

Another method for visualizing airway obstruction involves conducting cine magnetic resonance imaging under sedation, which facilitates a three-dimensional assessment of the upper airway. This technique proves advantageous in surgical planning for pediatric patients with potential multiple obstruction sites, attributed to obesity, craniofacial syndromes, or neuromuscular disorders, as well as in children presenting with persistent OSA following ATE [42].

Non-surgical treatment options for residual OSA in children include several approaches. In mild residual OSA with minimal symptoms, observation without immediate intervention may be appropriate [43]. Obesity is a significant risk factor for OSA, and therefore weight management is important in obese children, as it can lead to significant improvements or the resolution of OSA [44]. Treatment options also include medication, particularly intranasal corticosteroids, and leukotriene modifiers, which may help in mild OSA accompanied by nasal obstruction [45].

Rapid maxillary expansion (RME) is an orthodontic procedure that widens the palate and nasal passages, improving airway patency in children with a narrow palate and residual OSA [34,46].

For children with persistent OSA due to causes other than adenotonsillar hypertrophy, such as obesity or Down syndrome, additional surgical procedures may be necessary. These include lingual tonsillectomy and tongue base procedures, which use minimally invasive techniques to reduce obstructions. Midline posterior glossectomy and tongue base advancement are other surgical options that can significantly reduce the AHI, especially in children with Down syndrome [47].

Hypoglossal nerve stimulator is an implantable device approved for children with Down syndrome over the age of 13 that stimulates the nerve and prevents airway collapse during sleep [48,49]. Overall, the treatment of residual OSA in children is multidisciplinary and requires a combination of diagnostic and therapeutic approaches tailored to the individual profile of the child.

Managing residual pediatric OSA, especially in complex cases, requires a multidisciplinary approach. This includes collaboration between pediatric pulmonologists, sleep specialists, otolaryngologists, and, where necessary, orthodontists.

For children with genetic syndromes, craniofacial anomalies, or severe obesity, a more individualized treatment plan is necessary.

Nevertheless, our study is constrained by certain limitations, primarily the sample size of participants. The relatively small cohort of 34 patients limits the generalizability of our findings, as it may not fully capture the heterogeneity of the broader patient population with obstructive sleep apnea (OSA). A limited sample size also reduces the statistical power of the study, potentially hindering the detection of subtle but clinically relevant differences or associations. Additionally, with fewer participants, subgroup analyses become less reliable, limiting insights into how different phenotypes or patient characteristics influence outcomes. Future research with larger, more diverse cohorts is necessary to validate our results and provide more robust evidence that can be extrapolated to clinical practice. Despite these constraints, this study offers valuable preliminary data and highlights important trends regarding the incidence and severity of OSA before and after surgery. Future studies should focus on expanding the sample size and including additional potential factors influencing the persistence of OSA to improve the accuracy and reliability of predictive models.

Phenotyping is very important in pediatric OSA because it allows better understanding of the various clinical manifestations, risk factors, and treatment responses in children. Identifying specific phenotypes helps to manage the disease more precisely and predict the course and risk of residual OSA after adenotonsillectomy or other interventions. However, a validated phenotyping tool that systematically and reliably classifies pediatric patients into relevant clinical groups is still lacking in the pediatric population. We consider this area essential for further progress in the diagnosis and therapy of pediatric OSA and emphasize the need to develop and validate standardized phenotyping tools that would enable an individualized approach in clinical practice.

The significance of sleep during childhood should not be underestimated and must be integrated into routine medical anamnesis. Parents should be reminded that even if symptomatic relief and snoring cessation occur post-surgery, residual obstructive apnea might persist. Therefore, the execution of follow-up polysomnography and ongoing consultation with a pediatric somnologist is crucial. Effective management of pediatric patients afflicted by obstructive and residual obstructive sleep apnea through targeted and individualized treatment markedly enhancs the quality of life for the child and their family while also averting the continuance of significant clinical repercussions of this condition into adulthood.

## 5. Conclusions

While ATE remains the first-line treatment for pediatric OSA, a significant number of children may continue to experience residual OSA postoperatively. A thorough postoperative evaluation with PSG, including consideration of additional therapies and the potential use of DISE, can help to refine treatment and improve outcomes for children with persistent OSA. Differentiating phenotypes in each patient may lead to more targeted diagnostic approaches and help identify subgroups more likely to respond to specific treatments, playing a critical role in the development of personalized medicine strategies for pediatric OSA patients.

## Figures and Tables

**Table 1:** Gender distribution in entire cohort and in each phenotype.

	TOTAL	Common Phenotype	Adult Phenotype
	34	14	20
Male	29 (85.3%)	12 (85.7%)	17 (85.0%)
Female	5 (14.7%)	2 (14.3%)	3 (5%)

**Table 2:** Relationship between age, z-score, AHI and phenotypes.

	TOTAL	Common Phenotype	Adult Phenotype	*p*
*n*	34	14	20	-
AGE	9.2 ± 4.4	6.1 ± 3.1	11.4 ± 3.8	*p* < 0.001
BMI z-score	1.42 ± 1.62	−0.29 ± 0.83	2.61 ± 0.67	*p* < 0.001
AHI	32.2 ± 31.9	40.2 ± 34.9	27.1 ± 20.5	*p* = 0.253

**Table 3:** Incidence of residual OSA in individual phenotypes.

	Common Phenotype	Adult Phenotype
With residual OSA	8 (57.1%)	16 (80.0%)
Without residual OSA	6 (42.9%)	4 (20.0%)

**Table 4:** Incidence and severity of OSA before and after surgery in our cohort of 34 patients.

Patients	Before Surgery	After Sugery
Without OSA	0	10 (29.4%)
Mild OSA	0	11 (32.4%)
Moderate OSA	6 (17.6%)	7 (20.6%)
Severe OSA	28 (82.4%)	6 (17.6%)

## Data Availability

The original contributions presented in this study are included in the article. Further inquiries can be directed at the corresponding author.

## Abbreviations

The following abbreviations are used in this manuscript:

AASM	American Academy of Sleep Medicine
AHI	Apnea-Hypopnea Index
BMI	Body Mass Index
BiPAP	Bilevel Positive Airway Pressure
CPAP	Continuous Positive Airway Pressure
DISE	Drug induced sleep endoscopy
NREM	Non-Rapid Eye Movement
OSA	Obstructive Sleep Apnea
OSAS	Obstructive Sleep Apnea Syndrome
PSG	Polysomnography
REM	Rapid Eye Movement
RME	Rapid maxillary expansion
SPT	Sleep Period Time
TIB	Time in Bed
TST	Total Sleep Time
WASO	Wake After Sleep Onset
